# Multi-Drug Resistance Transporters and a Mechanism-Based Strategy for Assessing Risks of Pesticide Combinations to Honey Bees

**DOI:** 10.1371/journal.pone.0148242

**Published:** 2016-02-03

**Authors:** Alex J. Guseman, Kaliah Miller, Grace Kunkle, Galen P. Dively, Jeffrey S. Pettis, Jay D. Evans, Dennis vanEngelsdorp, David J. Hawthorne

**Affiliations:** 1 Department of Entomology, University of Maryland, College Park, Maryland, United States of America; 2 Department of Chemistry, University of North Carolina, Chapel Hill, North Carolina, United States of America; 3 Bee Research Laboratory, United States Department of Agriculture–Agricultural Research Service, Beltsville, Maryland, United States of America; University of British Columbia, CANADA

## Abstract

Annual losses of honey bee colonies remain high and pesticide exposure is one possible cause. Dangerous combinations of pesticides, plant-produced compounds and antibiotics added to hives may cause or contribute to losses, but it is very difficult to test the many combinations of those compounds that bees encounter. We propose a mechanism-based strategy for simplifying the assessment of combinations of compounds, focusing here on compounds that interact with xenobiotic handling ABC transporters. We evaluate the use of ivermectin as a model substrate for these transporters. Compounds that increase sensitivity of bees to ivermectin may be inhibiting key transporters. We show that several compounds commonly encountered by honey bees (fumagillin, Pristine, quercetin) significantly increased honey bee mortality due to ivermectin and significantly reduced the LC_50_ of ivermectin suggesting that they may interfere with transporter function. These inhibitors also significantly increased honey bees sensitivity to the neonicotinoid insecticide acetamiprid. This mechanism-based strategy may dramatically reduce the number of tests needed to assess the possibility of adverse combinations among pesticides. We also demonstrate an *in vivo* transporter assay that provides physical evidence of transporter inhibition by tracking the dynamics of a fluorescent substrate of these transporters (Rhodamine B) in bee tissues. Significantly more Rhodamine B remains in the head and hemolymph of bees pretreated with higher concentrations of the transporter inhibitor verapamil. Mechanism-based strategies for simplifying the assessment of adverse chemical interactions such as described here could improve our ability to identify those combinations that pose significantly greater risk to bees and perhaps improve the risk assessment protocols for honey bees and similar sensitive species.

## Introduction

Annual losses of honey bee colonies, including overwintering losses, remain high, ranging between 34 and 45% in recent surveys [1, 2]. Various factors have been proposed to explain losses, including parasites and pathogens (particularly the parasitic mite *Varroa destructor*), poor nutrition, and pesticides [3]. The role of pesticides as a factor in increased mortality rates has received considerable attention [4–7]. Managed honey bee colonies are exposed to potentially dangerous pesticides in two distinct ways, through in-hive miticides to control parasites [7, 8] and through the foraging activity of adult bees who collect pollen and nectar which has been contaminated by environmental toxins, including agricultural pesticides [9, 10]. While there are notable examples of acute colony mortality associated with pesticide exposure, especially recently in planting-dust [11], by and large such events are rare, and the levels of individual pesticides found in colonies generally low [9, 10, 12]. Considered singly, insecticide exposures, inside and out of the hive, are typically below the levels thought to have large negative effects on colony health [13, 14]. The problem is that this expectation is based on sensitivity of bees to an insecticide in single-toxin bioassays. Single toxin exposures are not realistic and combinations of pesticides and in-hive medications, all at individually benign concentrations, may not have benign consequences. The routine treatment of bee hives with mixtures of acaricides and antibiotics, combined with the diversity of pesticides and secondary plant compounds that bees encounter, collect and concentrate in their hive, may pose a greater threat to bees than predicted from their responses to the individual compounds [7, 8, 15, 16]. To accurately assess the risk posed by pesticides, the synergistic, antagonistic and/or additive effects of the many xenobiotic chemicals bees encounter must be considered.

Because honey bees are exposed to many different xenobiotics it is impractical to test combinations of all of them to assess the likelihood of adverse interactions. Mullin et al. [6] found over 121 different pesticides in surveyed colonies; if we were to limit testing of only 2-compound interactions among these 121 pesticides, more than 7,000 tests would be needed to test all possibilities. If, however, the problem is simplified by testing the effect that pesticides have on those key metabolic and transport-excretion processes that drive the dynamics of toxin distribution, metabolism and excretion in bees, we may better predict which compounds are likely to participate in an adverse synergistic interaction at the individual bee and possibly colony level [17]. This mechanism-based approach is used in pharmaceutical regulation to identify potentially dangerous drug-drug and drug-food combinations, focusing on metabolizing enzymes such as cytochrome P450 (Cyp) enzymes and xenobiotic-handling proteins belonging to two transporter superfamilies, the ATP Binding Cassette (ABC) and the Solute Carrier transporters. These mechanisms are targeted because they interact with a wide variety of drugs and plant compounds and are likely causes of adverse interactions among them [18, 19]. Johnson et al. [7, 20] have discussed the importance of the Cyp enzymes in honey bees, but the roles of transporters have been neglected. Transporters mediate the movement of xenobiotics through membranes, expediting the movement of toxins and metabolites towards excretion and preventing infiltration of sensitive tissues by those compounds. Certain ABC transporters, in particular the ABC-B (MDR or Multi-Drug Resistance transporters), ABC-C (MRP or multidrug-resistance associated proteins) and ABC-G subfamilies are likely responsible for multiple chemical interactions in honey bees, because they have diverse substrate ranges including many pesticides and plant produced chemicals [21–25]. Because we are currently unable to functionally distinguish these subfamilies in honey bees, we refer here to them collectively as “MDR transporters”.

An adverse interaction of two chemicals encountered by bees would occur through the inhibition of key metabolic enzymes or transporters by one compound that subsequently reduces that protein’s capacity to process subsequent, possibly different, toxic substrates. We propose methods for determining if a xenobiotic inhibits the function of key transporters. Testing new or in-use pesticides in combination with standard substrates of detoxification mechanisms can identify those compounds that inhibit or increase the function of that mechanism. Similarly, tests using standard inhibitors of these xenobiotic-handling mechanisms can identify which xenobiotics may be substrates of that mechanism. Substrates and inhibitors of ABC transporters are known from work in mammalian, invertebrate and microbial systems [26–29] and are a good starting point for finding xenobiotic substrates and inhibitors of those transporters in bees. Hawthorne and Dively [8] show, for example, that inhibition of honey bee MDR transporters significantly increases honey bee sensitivity to two acaricides (fluvalinate and coumaphos respectively) indicating that they are substrates of those transporters in honey bees. Previously, Bain et al. [29] had identified fluvalinate and coumaphos as substrates and/or inhibitors of the key MDR transporter P-glycoprotein (P-gp). Both fluvalinate and coumaphos have been widely applied to bee colonies for control of the honey bee parasite *Varroa destructor*, and remain among the most common pesticide residues found in colony matrices [6, 30]. In another example, Schrickx and Fink-Gremmels [31] identified oxytetracycline as an inhibitor and/or substrate of mammalian P-gp. This antibiotic, which is applied to bee colonies for prophylactic treatment of the disease American foulbrood, increased honey bee sensitivity to fluvalinate and coumaphos, as would be expected if the transporters performed similarly in mammalian and hymenopteran systems [8].

Two types of assays are typically used for *in vivo* study of xenobiotic transporter function; inhibitor assays that sensitize cells or individuals to toxic substrates through chemical disruption of transporter function and labelled substrate assays which track the differential movement of substrate compounds in the presence and absence of inhibitors. Inhibitor assays are relatively easy to perform on honey bees and their endpoints (often mortality or dysfunctional behavior) are easily interpreted. However, even for well-characterized inhibitors and substrates, it remains possible that they affect more than one detoxification or excretion process. A complementary labeled-substrate assay(s) could help confirm the specificity of an inhibitors effect.

Here we investigate the use of ivermectin as a standard substrate for assessing the function of MDR transporters in honey bees. Ivermectin is an anthelminthic and acaricidal medication, with human and veterinary applications. It is known to interact with the multi-drug resistance (MDR) transporters in the ABC-B and/or ABC-C families of xenobiotic transporters [26, 32–34]. Ivermectin is a semisynthetic macrocyclic lactone derived from fermentation products of *Streptomyces avermitilis* [35] and it targets the glutamate-gated, and to a lesser degree the GABA-gated chloride channels of the insect nervous system [36, 37]. Although ivermectin is not applied widely for pest control in crops, several important insecticides, acaricides and nematicides, such as abamectin and emamectin benzoate, share ivermectin’s structural features and target sites [35]. Abamectin resistance in Drosophila has been shown to be strongly related to P-gp expression and function [38]. The interaction of MDR transporters with ivermectin was first noted when a strain of mice lacking the ABC-B transporter P-gp, died following ivermectin treatment for parasites [33]. Increased MDR transporter function is also known to contribute to ivermectin resistance in parasitic nematodes, cattle ticks, and head lice [39–42]. Silencing those transporters via RNAi reverses ivermectin resistance in lice [41], further supporting observations that xenobiotic-transporting ABC transporters mediate the sensitivity of arthropods to ivermectin.

We also test the inhibitory effects of several compounds on honey bee MDR transporters by measuring changes in honey bee sensitivity to ivermectin after exposure to test compounds. Ivermectin is toxic to honey bees, so we expect that co-exposure of ivermectin with an MDR transporter inhibitor will significantly increase sensitivity to this toxin.

MDR transporters may not act alone to protect bees from ivermectin toxicity. Bees may also use metabolic enzymes such as esterases and CYP enzymes to metabolize the toxin. Therefore changes in abundance of those enzymes could also alter honey bees sensitivity to ivermectin [36]. If ivermectin toxicity is indeed mediated by more than one process in bees, its utility as a model substrate for identifying candidate inhibitory compounds would be enhanced, at the expense of knowing which process was most responsible.

In this study, we first assess the dose effect of a standard inhibitor of MDR transporters, verapamil, on honey bee sensitivity to ivermectin. Verapamil is known to inhibit vertebrate MDR transporters and in insects has been shown to slow the transmembrane transport of P-gp substrates across the blood-brain barrier and across Malphigian tubule epithelia [43–45]. It has been suggested, at least for vertebrate cell lines that as an L-calcium channel blocker verapamil may contribute to increased sensitivity to xenobiotics in other ways as well [46]. We then test three compounds, previously shown to interact with MDR transporters in other organisms, for their potential to synergize ivermectin toxicity; fumagillin, an anti-microbial compound used to treat honey bee hives for the intestinal parasite *Nosema sp*., Pristine, a crop fungicide composed of the two active ingredients boscalid and pyraclostrobin used on fruit and nut trees during bloom, and quercetin, a plant compound found in pollen and nectar. Fumagillin and quercetin are known to inhibit the function of mammalian P-gp [47, 48] and boscalid and pyraclostrobin resistance in the fungal plant pathogen botrytis, has been attributed in part to changes in the MDR transporters of that pathogen, suggesting that the fungicides interact with those transporters in botrytis [49]. Although bees are not frequently exposed to ivermectin, any indication that these test compounds inhibit the function of the MDR transporters of bees by increasing their sensitivity to ivermectin would suggest that exposure to these compounds may sensitize bees to more common chemicals in the bee environment.

In addition to testing the effects of fumagillin and quercetin on honey bee sensitivity to ivermectin, we also tested these compound’s effects on honey bee sensitivity to the neonicotinoid insecticide acetamiprid. We have previously shown that bees are sensitized to acetamiprid following ingestion of verapamil, suggesting that acetamiprid is a substrate of those transporters in honey bees [8]. A commonly encountered environmental chemical that inhibits MDR transporters and increases sensitivity of bees to both ivermectin and acetamiprid, could similarly increase bee’s sensitivity to a broad array of compounds that are also substrates of MDR transporters posing a risk to hive health under field conditions.

The use of bioassays with well-characterized inhibitors and substrates is complemented by labeled-substrate assays of transporter function. Rhodamine B (RhB) is a fluorescent substrate of P-gp and possibly other MDR transporters. RhB is used to assess transporter function in many organisms including insects, bivalves, and fish [50–52]. Given a simple model of RhB dynamics within a honey bee following orally ingestion of this fluorophore, we expect MDR transporters in the midgut epithelium to slow the rate of RhB movement into hemolymph and for those transporters in the Malpighian tubules to remove RhB from hemolymph. Therefore, we expect hemolymph concentrations of RhB to be higher in bees treated with transporter inhibitors as a result of reduced transporter function in the midgut and Malpighian tubules [45, 53–56]. We also expect higher RhB concentrations in the heads (composed largely of brain and salivary glands) of bees treated with inhibitors because, in addition to higher hemolymph-mediated exposure, these transporters have key roles in maintaining the blood-brain barrier in insects, preventing the accumulation of xenobiotics in brain tissue [43, 44, 50].

## Materials and Methods

### Honey Bees

*Apis mellifera* were obtained from hives maintained on the University of Maryland farm in Beltsville, MD. Hives were maintained by standard bee keeping practices, and kept free of in-hive medications. Brood frames were taken from hives and emerging bees were collected daily into 207 ml cages of 15–20 bees and fed 30% sucrose w/v solution. Bees used in bioassays were 3–7 days post-eclosion.

### Chemicals

Ivermectin (>95%) was purchased from MP Biomedicals, (Solon, OH), verapamil, quercetin, Rhodamine B were purchased from Sigma, a formulated solution of acetamiprid (Assail) was a gift from United Phosphorous Inc. (King of Prussia, PA), and formulated ‘Pristine’ was a gift of BASF Corp. (Research Triangle Park, NC). Ivermectin, the candidate inhibitors, and verapamil were fed to bees in a 30% sucrose solution. Quercetin was first dissolved in DMSO in a 50 mM solution before dilution to 1 mM in a 30% sucrose solution. All other inhibitors were dissolved in distilled water. Because Pristine is a formulation containing two fungicides, boscalid and pyraclostrobin, a 50 mM stock was made using the combined molecular weights of the components weighted by their relative fraction of the total mass in the formulation.

### Inhibitor bioassays

Bees were fed (*ad lib*.) 1 mM solutions of verapamil, fumagillin, Pristine, or quercetin for 24 h (in 30% sucrose syrup) and then fed sucrose solutions containing 1 mM of the same test inhibitor and 1 μg/ml ivermectin. Mortality was recorded 24 h after ivermectin exposure for 20–28 replicate cages of bees with approximately 15 bees in each cage. No-inhibitor controls (15 cages) were included in each assay. Subsequently, using the same bioassay protocol, a series of 6 concentrations of ivermectin were used to estimate shifts in the LC_50_ of ivermectin resulting from ingestion of the 1 mM inhibitor. Mortality rates for each concentration of ivermectin were estimated by averaging the mortality of approximately 15 bees contained in each of 4 replicate cages. Similarly, to measure changes in sensitivity to ivermectin at varying dosages of the inhibitors, 4–12 replicate cages of bees (fewer at extreme dosages, more at intermediates) were fed a series of concentrations of each inhibitor (0, 0.03, 0.06, 0.125, 0.25, 0.5, 1.0, 2.0 mM) for 24 h, then fed a combination of the same inhibitor dose and 1μg/ml ivermectin solution.

Changes in sensitivity to the neonicotinoid insecticide acetamiprid following pre-treatment of bees with the inhibitors verapamil, fumagillin and quercetin (1 mM) were tested using a 0.1 μg/μl concentration of the active ingredient in formulated insecticide. Mortality rates for groups of 5 to 15 bees were estimated by averaging results from12 replicate cages for each inhibitor–acetamiprid combination. Mortality of bees in 3 cages for each inhibitor–without insecticide were also recorded.

#### Analysis

Mortality was estimated as the proportion of bees within a cage that were dead at 24 or 48 h after treatment with ivermectin. Comparisons of mean mortality among treatments were tested using t-tests. LC_50_s of ivermectin with inhibitors were estimated using the ProcProbit procedure in SAS (V. 9.2).

### Functional Transport Assays

Cohorts of 10–15 bees in mesh-covered cages (207 ml) were fed 30% sucrose solutions containing a range of verapamil concentrations (0, 0.05, 0.1, 0.33, 0.66, 1.0 mM) for 24 h. Cohorts were then shallowly anesthetized by chilling. During recovery, bees were hand-fed 10 μl of RhB (0.125 mg/ml) in 30% sucrose syrup, then placed individually into a 15 mL centrifuge tube for 2 h to allow each bee to fully consume the solution. Bees were then returned to their original cups along with similarly treated cohort members and continued feeding on their assigned inhibitor treatment *ad lib*.

Concentrations of RhB were measured in hemolymph, heads, and whole guts from each of 8–15 anesthetized bees, 24 or 48 h after dye consumption. Hemolymph concentrations of RhB were estimated from 1 μl samples of hemolymph collected from individual bees. Hemolymph was added to 50 μl 0.01% SDS, mixed thoroughly and transferred to a 96 well plate for measurement of RhB fluorescence. Rhodamine B concentrations in the head were measured by removal of the head from the body, removal of mandibles and proboscis, and dicing the head into small pieces with dissecting scissors. The diced tissue was pestle-macerated in a 1.5 ml tube with 50 μl 0.01% SDS. Into each tube, 200 μl 0.01% SDS and four stainless steel beads (two 1.4 mm and two 2.3 mm) were added and the tubes vortexed for 4 minutes at 3000 RPM with a 0.5 second pulse every 2 s with a Vortex Genie Pulse (Scientific Industries, Bohemia, New York). Tubes were centrifuged for 240 s at 5000 RPM and RhB fluorescence measured from 50 μl of supernatant. The entire gut of the bees, including Malpighian tubules, was dissected and crushed with a pestle in a 1.5 ml tube containing 50 μl 0.01% SDS. After addition of 100 μl 0.01% SDS, each sample was vortexed for 5 s before centrifugation (240 s, 5000 RPM) and analysis of the supernatant solution (50 μl) for RhB fluorescence. Fluorescence of all samples was measured using a Molecular Devices FilterMax F5 spectrophotometer (excitation 535 nm, emission 595 nm).

#### Analysis

To correct for variation in dye consumption by individual bees, fluorescence measures from hemolymph or head samples of an individual bee were indexed as a fraction of the combined fluorescence of all three tissues (hemolymph + head + gut) of that bee, relative to the mean of that variable for the control samples analyzed on the same day.

Tests of the relationship of verapamil concentration fed to bees and the RhB concentrations in the hemolymph and head tissues were performed using ProcMixed in SAS (V. 9.2). Because variances differed for those bees treated with low (0.01, 0.05 mM) versus high (0.33, 0.66, 1.0 mM) doses of verapamil, the covariance matrices for those data were estimated separately using a repeated statement.

## Results

### Bioassay tests of inhibitors

**Fig 1:**
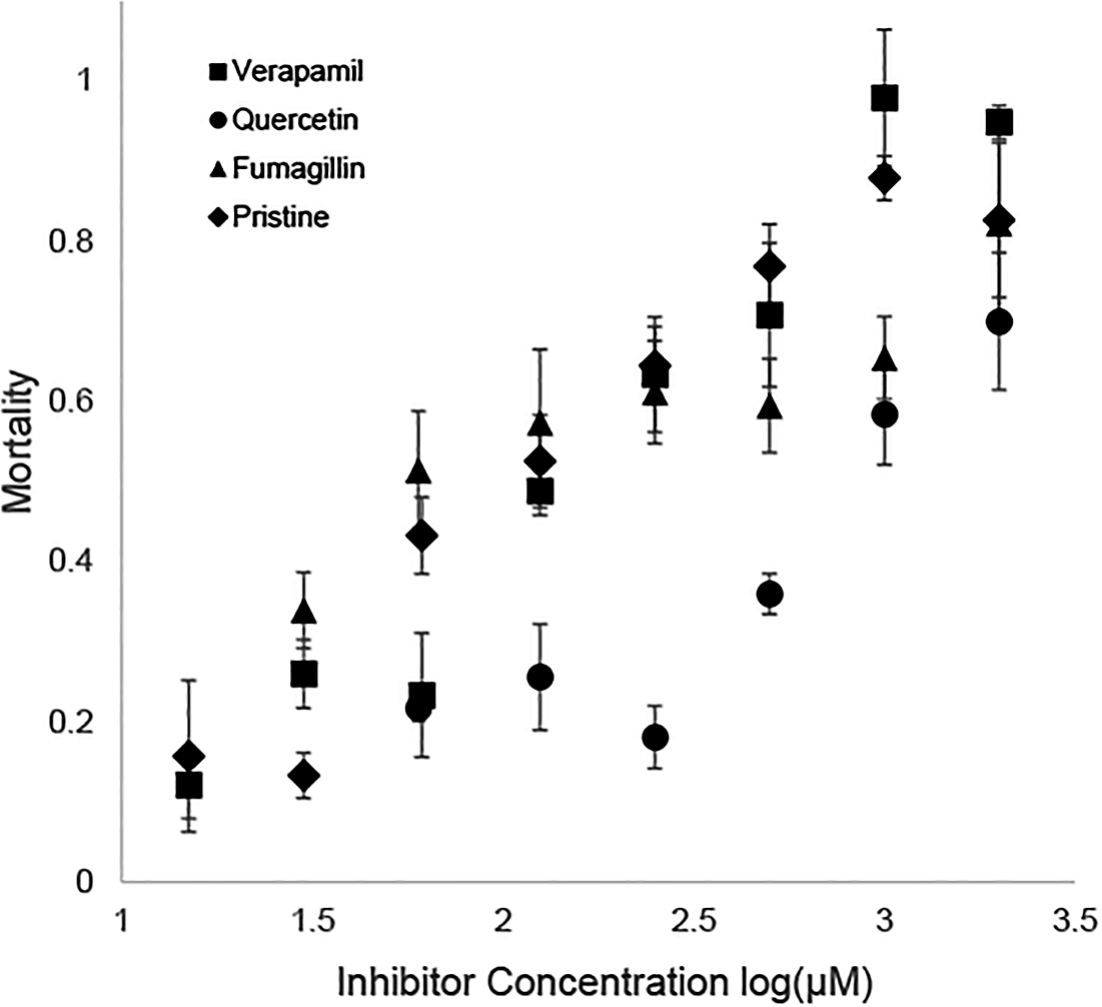
Mortality of honey bees (24 h ±SE) pretreated with a series of concentrations of candidate inhibitors. Bees were fed verapamil, quercetin, fumagillin or Pristine before and during oral exposure to 1 μg/ml ivermectin.

The 1 μg/ml concentration of ivermectin caused a mean mortality of 0.14 over numerous trials; significantly greater (p < 0.01) than the sucrose syrup control (0.04) but low enough to demonstrate additional sensitivity caused by candidate synergists. Verapamil-treated bees were significantly sensitized to 1 μg/ml ivermectin, with mean mortality increasing to 0.85, as were bees co-exposed to Pristine (0.82), fumagillin (0.47) and quercetin (0.54) (Fig 1). Inhibitor-only controls had very low mortality of 0.05, 0.05, 0.01 and 0.02 for verapamil, Pristine, fumagillin and quercetin, respectively (Table 1).

**Table 1:** Increased sensitivity of honey bees to ivermectin (1ug/ml) following oral exposure to standard and tested inhibitors (1mM) and LC50 of ivermectin following treatment of bees with 1 mM inhibitor solution. All inhibitor + ivermectin treatments had significantly higher mean mortality than the ivermectin treatment (p < 0.0001).

Treatment	Mortality (SE) 1 μg/ml IVM and 1mM Inhibitor	LC_50_ (μg/ml)	95%CI	Synergism Ratio (LC_50_ relative to IVM)
Ivermectin (IVM)	0.14 (0.02)	1.57	1.38–1.80	1.00
IVM + Verapamil	0.85 (0.03)	0.38	0.01–0.67	4.13
Verapamil	0.05 (0.02)			
IVM + Pristine	0.82 (0.03)	0.57	0.34–0.74	2.75
Pristine	0.05 (0.03)			
IVM + Fumagillin	0.47 (0.04)	0.86	0.47–1.25	1.83
Fumagillin	0.01 (0.01)			
IVM + Quercetin	0.54 (0.05)	0.61	0.41–0.77	2.57
Quercetin	0.02 (0.02)			

**Fig 2:**
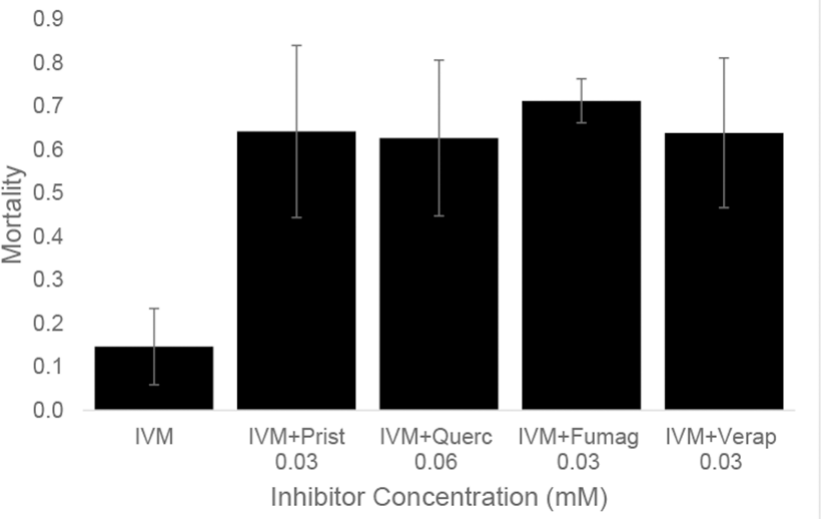
Mean 24 h mortality (±SE) of honey bees fed 1 μg/ml ivermectin treated with low concentrations of inhibitors. Bees fed Pristine (Prist), quercetin (Querc), fumagillin (Fumag) and verapamil (Verap) in sucrose syrup. All inhibitor/doses shown had significantly greater mortality than the ivermectin-only control (P < 0.002).

The LC_50_ estimate for bees fed ivermectin laced syrup after 24 h was 1.57 μg/ml. Using non-overlapping 95% confidence intervals as a guide for statistical significance, all of the candidate inhibitors caused significant shifts towards increased sensitivity of tested bees to ivermectin (Table 1), with synergism ratios of 2–4 fold for 1 mM inhibitor. Additional assays using a series of 2-fold dilutions of the inhibitors resulted in gradual declines in sensitivity to 1 μg/ml ivermectin (Fig 1), with the lowest concentrations (0.03 or 0.06 mM) showing significantly greater mortality than ivermectin-only treatments for all 4 inhibitors (Fig 2).

#### Acetamiprid assay

**Fig 3:**
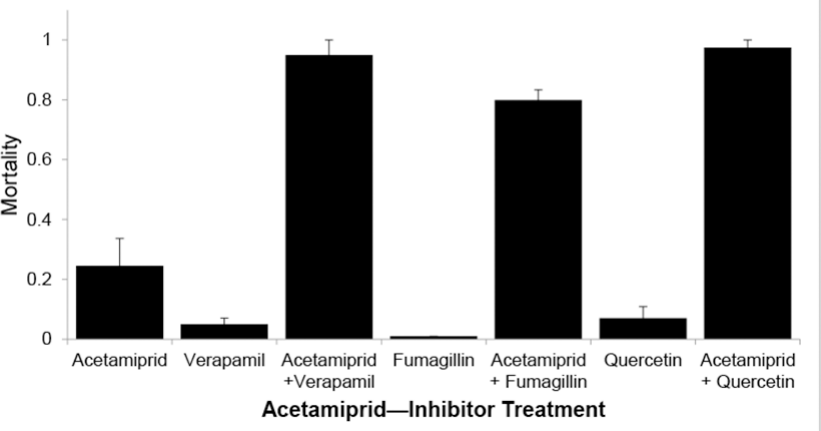
Increased sensitivity of honey bees to the neonicotinoid insecticide acetamiprid. Mean mortality (±SE) of honey bees fed 0.1 μg/μl acetamiprid with 1 mM solutions of inhibitors in sucrose syrup. All inhibitor-acetamiprid combinations had significantly greater 24 h mortality than the acetamiprid-only control (P < 0.0002).

Sensitivity to acetamiprid (0.1 μg/μl) was increased by 3 of the inhibitors (Fig 3) and the increases in mortality were similar to those observed for ivermectin. Control mortality from acetamiprid alone at that concentration was 0.25. We observed increased mean mortality to 0.95, 0.80 and 0.97 when bees were treated with verapamil, fumagillin and quercetin, respectively (Fig 3). Mean mortality was very low for 3 replicate cages of bees fed only verapamil (0.05), fumagillin (0.01) or quercetin (0.07).

### Functional assay of transporters using RhB dye

**Fig 4:**
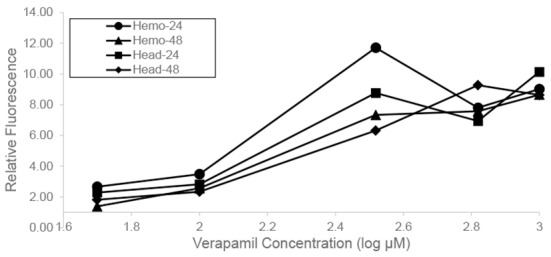
Rhodamine B fluorescence of head and hemolymph tissues. Mean fluorescence relative to no-verapamil controls (24 and 48 h post feeding) at a series of verapamil concentrations.

There was a significant increase in hemolymph and head RhB concentrations with increasing dosages of verapamil after both 24 and 48 h (Table 2, Fig 4). Tissues from verapamil-treated bees had 2.66–11.72 fold higher hemolymph and 2.28–10.13 fold higher head RhB concentrations than control bees after 24 h (Fig 4). Mean RhB concentrations in hemolymph were significantly lower after 48 h when compared to 24 h measures (Table 2). There was a similar, but non-significant trend for RhB concentration in head tissue. It may be that RhB is cleared from the hemolymph more rapidly than brain tissue, perhaps through the normal function of the Malpighian tubules resulting in reduced RhB in hemolymph after 48 h.

**Table 2:** Transporter function assays. Bees treated with a series of dosages of the MDR transporter-inhibitor verapamil before and after ingestion of the fluorescent substrate Rhodamine B solution, and assayed at two times (24 and 48 h post ingestion) show greater fluorescence at increased verapamil concentrations in both tissues.

**Tissue**	**Effect**	**df**	**F**	**P-value**
**Hemolymph**	Time	1,83	5.57	0.021
	Dose	1,83	61.51	<0.0001
	Dose × Time	1,83	0.07	0.80
**Head**	**Effect**	**df**	**F**	**P-value**
	Time	1,83	1.10	0.30
	Dose	1,83	78.21	<0.0001
	Dose × Time	1,83	0.05	0.82

## Discussion

We show that ivermectin is an effective test-substrate for identifying compounds that inhibit the function of MDR transporters in honey bees by sensitizing bees to ivermectin with the MDR transporter-inhibitor verapamil. This interaction has previously been observed with mosquitoes [57], parasitic nematodes [39], cattle ticks [58] and human body lice [41]. We also show that reductions in the function of MDR transporters can be measured by increased accumulation of the fluorescent substrate RhB in hemolymph and head tissues of bees, similar to previously observations in Drosophila, aquatic insects, mollusks, and many cell-transport assays [50, 59, 60].

Many agricultural pesticides are known to interact with MDR transporters as substrates or inhibitors including members of most classes of insecticides and fungicides [21]. We tested several compounds that honey bees commonly encounter, all previously shown to interact with MDR transporters in other systems, and found them to increase bee’s sensitivity to ivermectin and the neonicotinoid insecticide acetamiprid. We are especially interested in the MDR transporters here because their substrate promiscuity will likely cause numerous adverse interactions among xenobiotics in bees’ environments when the transporters are inhibited. Ivermectin served here as an effective model substrate for analysis of MDR transporter-mediated inhibition of detoxification and excretion systems in honey bees. The increased sensitivity of bees to ivermectin following exposure to Pristine, fumagillin, and quercetin is evidence that those compounds are interacting with the MDR transporters in bees to synergize the toxicity of ivermectin, although other mechanisms cannot be excluded. These inhibitors may similarly sensitize bees to other xenobiotics that are substrates of these transporters. Indeed, fumagillin, quercetin and verapamil each sensitized bees to the neonicotinoid insecticide acetamiprid, a toxin previously shown to be a substrate of the MDR transporters in bees [8]. It is important to note that because ivermectin is not a crop pesticide, it is not a significant threat to honey bees, although two similar insecticides (abamectin and emamectin benzoate) are agricultural/forestry insecticides and warrant testing. In most of our bioassays candidate inhibitors were tested at concentrations higher than those likely experienced by bees in the field. However, trials of much lower, possibly field-relevant (ca. 0.05x) concentrations of the inhibitors show that they continue to increase honey bee sensitivity to ivermectin (Fig 2). Similarly, the dosage of acetamiprid used here is greater than that typically encountered by foraging bees and any conclusions about honey bee risk to acetamiprid exposure requires additional research. Our objective here is simply to assess the suitability of ivermectin as a surrogate for acetamiprid and perhaps other formulated pesticides that are, despite very different chemistries and target sites, also substrates of honey bee MDR transporters.

Fumagillin, Pristine and quercetin were tested here because they have previously been shown to interact with MDR transporters and they are commonly found in a bees’ environments. The observation that these compounds synergize the toxicity of ivermectin and acetamiprid suggests that they pose risks for bees when exposed to other xenobiotics. Fumagillin is widely used to treat and prevent *Nosema sp*. infection of bees in North America, but its use is not permitted in the EU [61]. It is applied to hives in the fall and again in the spring, often in a heavy sugar syrup solution [62]. Although the 1 mM concentration used in most of our experiments is higher than the label-rate field exposures of bees (field rates estimated from label are 0.054 mM), bees may concentrate and store the fumagillin-laced syrup as honey resulting in a prolonged exposure at a range of concentrations. Dosages of fumagillin as low as 0.03 mM, clearly within the range of expected exposures of treated bee hives, increase sensitivity of bees to ivermectin (Fig 2). Because fumagillin is widely used, most commonly by commercial bee keepers, and is likely an inhibitor of the MDR transporters in bees, it may alone or in combination with other inhibitors create significant sensitivity to otherwise tolerable dosages of pesticides or other toxins which are substrates of the transporters. Johnson et al. [63] also observed increased honey bee sensitivity to tau-fluvalinate following treatment with fumagillin. The seasonal pattern of fumagillin hive treatments coincides with application of oxytetracycline, also shown to sensitize bees to pesticides; also potentially through inhibition of MDR transporters [8]. Further testing is required to confirm that field-relevant dosages of fumagillin have a significant impact on sensitivity to field-relevant concentrations of pesticides.

Pristine is a fungicide blend containing two active compounds and other ingredients. Pristine is commonly applied at blossom to many crops including almonds and tree-fruits, assuring honey bee exposure. Residues of the component fungicides have been recovered from hives [5, 64], but increased mortality of bees exposed to Pristine alone has not been reported. Johnson et al. [63] showed a 3-fold synergism of the pyrethroid tau-fluvalinate following exposure to the components of Pristine. Because fluvalinate is likely a substrate of the MDR transporters [8, 29], this interaction may be due to a competitive inhibition of the transporters by the fungicides. Johnson et al. [63] tested the components of Pristine (pyraclostrobin and boscalid) separately and found that both sensitized bees to fluvalinate, pyraclostrobin more so. Additional testing needs to be done on individual components of this pesticide formulation to determine which are causing the effects observed here, and to evaluate effects at dosages to which bees are exposed under field conditions. If Pristine or one of its components contributes to the inhibition of MDR transporters as we suspect from these data; the exposed bee colonies could become more sensitive to an array of pesticides that are substrates of the bees MDR transporters.

Quercetin is a commonly encountered secondary plant compound (flavonoid), found in nectar and pollen of many plants and in the propolis and honey of bee hives. It is an inhibitor of MDR transporters in assays of mammalian and insect cells and nematodes [65, 66] and it may also interact with Cyp metabolic enzymes [67]. The amount of quercetin in nectar and pollen varies among crops [68], suggesting that sensitivity of bees to toxic pesticides may vary with forage characteristics.

In addition to identifying some compounds that may increase honey bee sensitivity to insecticides, this study illustrates a strategy for streamlining the identification of dangerous combinations of chemicals among many possibilities, as is the case with honey bees. As an example, instead of testing candidate combinations one-by-one for adverse interactions among all of the potential candidates, we can now test an entire category of potential interactions (those mediated by MDR transporters) using increased sensitivity to ivermectin as an indication of MDR transporter inhibition. When assessing the potential for adverse interactions caused by multiple candidates, this strategy reduces the number of tests dramatically, screening pairwise interactions mediated by this mechanism in *N* tests for *N* candidate compounds instead of $\left(\frac{N*N-1}{2}\right)$ tests, a potentially very large difference. To test 10 compounds for adverse interactions with MDR transporters, one can test for those interactions in 10 instead of 45 tests, with additional testing of those showing significant synergistic effects if desired. If testing 100 compounds, it would be only 100 tests versus ca. 4,950 tests. This strategy substantially increases the practicality of assessing the risk of adverse interactions of combinations of agricultural chemicals to honey bees.

Clearly, metabolic mechanisms also mediate harmful pesticide interactions in bees and similar assays for the major classes of metabolic enzymes that may also have wide substrate ranges could be developed [63]. Interestingly, many pharmaceutical substrates of MDR transporters also interact with the Cyp metabolic enzymes [18, 19]. Therefore, use of ivermectin, or a similar substrate, as a test for transporter function, may identify compounds that also interact with metabolic enzymes.

Current assessment of pesticide risks to honey bees in the U. S. and Europe does not consider the altered sensitivity that would occur following exposure to combinations of pesticides. Given the diverse collection of compounds to which bees are exposed as a result of their foraging and food storage habits and veterinary treatments, it is clearly impractical to require assessment of a new product in combination with all other compounds that bees are likely to encounter. Mechanism-based strategies for simplifying the assessment of adverse chemical interactions such as described here could improve our ability to identify those combinations that pose significantly greater risk to bees and perhaps improve the risk assessment protocols for honey bees and similar sensitive species.
